# Measuring motivation for team science collaboration in health teams

**DOI:** 10.1017/cts.2020.567

**Published:** 2020-12-21

**Authors:** Gaetano R. Lotrecchiano, Lisa Schwartz, Holly J. Falk-Krzesinski

**Affiliations:** 1 Department of Clinical Research and Leadership, George Washington University School of Medicine and Health Sciences, Washington, DC, USA; 2 Department of Biomedical Laboratory Sciences, George Washington University School of Medicine and Health Sciences, Washington, DC, USA; 3 Elsevier, Global Academic Relations, New York, NY, USA; 4 Northwestern University School of Professional Studies, Chicago, IL, USA

**Keywords:** Team science, motivation, psychometrics, assessment, readiness

## Abstract

The Motivation Assessment for Team Readiness, Integration, and Collaboration (MATRICx) is a psychometric instrument that measures individual motivation for collaboration. It was validated using Rasch Analysis to create an indicator hierarchy on two dimensions: cooperation and collaboration. Six domains provide the basis for the tool to identify team member readiness for collaboration and a means by which to understand motivational strengths in a team based on degree of past self-reported experience. This brief report provides an overview of the development of the tool, how science teams may use it, and how to interpret results to advance team member readiness for greater collaboration. The paper also draws attention to ongoing work in progress to develop learning interventions to accompany the MATRICx instrument.

## Introduction

In an age when solving complex biomedical and health care problems necessitates sharing communal resources and developing multilateral networks among individual contributors, a clear understanding of the motivations and threats to forming successful collaborative teams is essential. Collaboration is a cooperative effort between two or more individuals striving towards a common goal [1] and is a major objective in many projects that seek to utilize novel and innovative techniques resulting from “boundary crossing” knowledge [2] and professional communities [3] with similar yet differently interpreted scientific goals. While the notion of collaboration is appealing, individuals still struggle with full engagement in multi- and interdisciplinary team enterprises [4,5]. Early thinking on collaboration readiness focused on intrapersonal factors in team science [6,7]. These inquiries attempted to ground readiness in the personal skills and competencies that might support group functioning. These individual skills and competency-based approaches have been utilized to measure group readiness in science and healthcare programs and include general measures such as willingness to embrace new technologies [8], readiness for organizational change [9], enhancing self-directed learning [10], and the role of digitally-mediated and interprofessional learning [11]. Those instruments that are more specifically oriented towards understanding Knowledge Producing Teams (KPTs) engagements focus more on understanding either ingroup interactions or measure readiness from the perspective of team output. Most notable of these types of assessments are those that measure attitudes toward transdisciplinary research and research collaborations [7], collaborative productivity [12], research orientation [13], perceptions of collaboration [14], psychological safety in teams [15], collective orientation [16], and leadership and employee creativity [17]. While motivators and threats to collaboration can be inferred from these types of studies, none directly assesses individual perceptions of the motivators and threats that draw professionals to form and work in teams highlighting the relationship between motivation and degrees of collaboration. This type of assessment requires a focus on the intrapersonal measures that are not the result of learned skills and behaviors but rather the pre-determinant antecedents that are often at the heart of why an individual may or may not be motivated to join and work in scientific teams [18]. While the reasons for this are numerous and can be applied to a number of mini-theories that contribute to the self-determination meta-theory, our approach to this question rests with basic psychological need theory, which posits that autonomy, competence, and relatedness are key aspects to promoting well-being and satisfaction of psychological needs [19]. In general, measuring skill competency continues to overshadow the measurement of individual motivations and threats to collaboration. As a response to this dominant trend, we have developed and validated the Motivation Assessment for Team Readiness, Integration, and Collaboration survey (MATRICx) to provide an inroad into understanding several key motivators to health science knowledge producing teams (KPTs) that have the ability to inform strategic workgroup engagement; sharing of knowledge through collaborative research and scholarship; and the creation of KPTs inclusive of multi-role and multi-discipline stakeholders.

## Materials and Methods

**Fig. 1.**
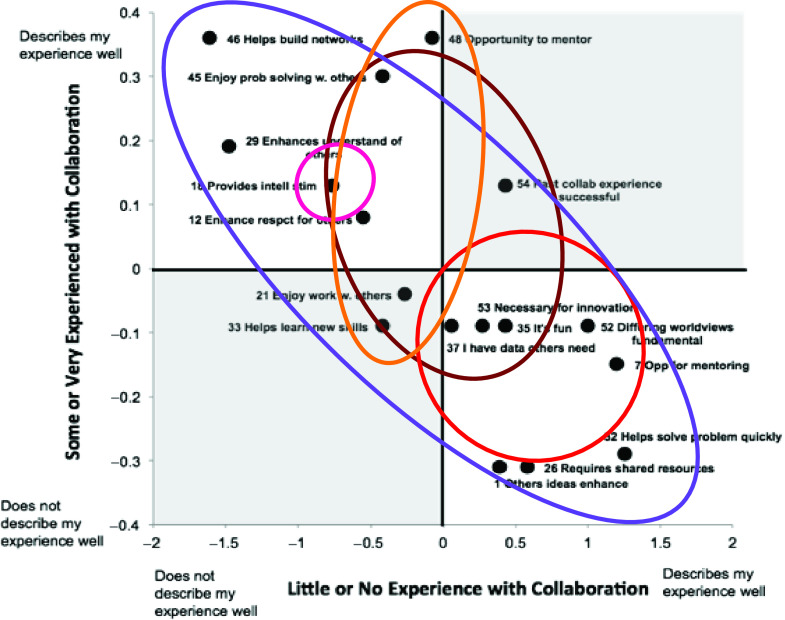
Hierarchy of MATRICx motivator items based on self-reporting of past experiences grouped by domain [25].

The development of the MATRICx began with an *ad hoc* set of 105 intrapersonal terms and phrases for motivation and threats to collaboration found in a select set of literature [20–23] and after conducting a comprehensive scoping review [24]. These indicators were reduced in number to 49 by identifying and deleting those indicators that overlapped in definition in the literature. The remaining indicators were subject to thematic analysis based upon the frequency upon which each indicator was reported in the literature by the same authors, keywords, and similar meaning, which resulted in the indicators being grouped into six domains (resource acquisition, recognition and reward, knowledge transfer, advancing science, building relationships, and maintenance of beliefs). The validation process included a three-phase series of pilot testing and intermittent cognitive testing using both Rasch analysis and a cognitive testing protocol that measured reliability of the items themselves [25]. Rasch analysis transforms ordinal data into log-odd probabilities (or logits), where a higher logit reflects more readiness to collaborate along a hierarchy. Rasch analysis also requires that items are related to a single underlying construct. Psychometric analysis [25] allowed for the reduction to only 17 motivator indicators that consistently fit into the same construct. The threats items did not ascribe to a parallel construct like the motivator items, leading the instrument to be primarily a measure of motivations. Using the Person Separation Index, we found that motivator items were better able to distinguish participants along the hierarchy (motivator items 0.9 *[1.7]*); (motivator items-experienced participants −0.4 *[0.4]*); and (motivator items-inexperienced participants 1.8 *[0.4]*), and thus we are able to measure collaborative readiness more precisely by focusing on motivations rather than including threats though they still make up a part of the survey. In addition, it was found that the motivator items aligned into a different hierarchy depending on past self-reported experience with working in teams. This finding led to the plotting of a hierarchy of motivators based on degree of self-reported past experience working in teams (Fig. 1). More details regarding the development and validation of the MATRICx can be found in previous reports [24,25].

**Fig. 2.**
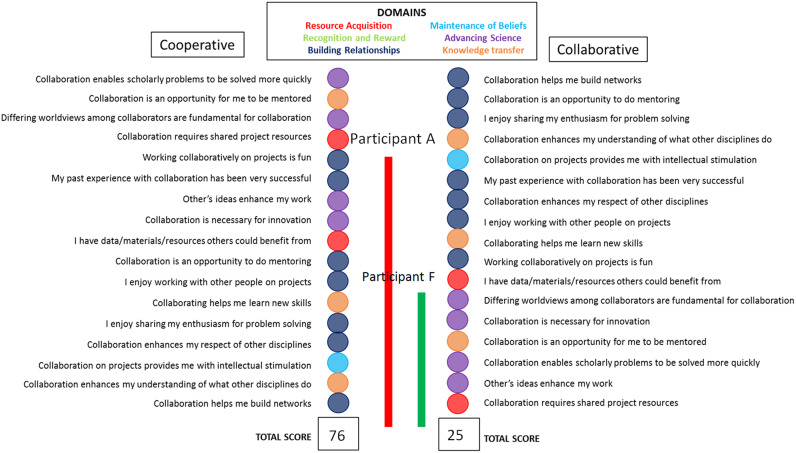
Hierarchy of items based on Cooperative and Collaborative Scales of Motivator Items.

Ultimately, the present MATRICx consists of only motivator items with two corresponding scales: cooperative (low formalization, low interdependence, autonomous groups share information to support each other’s organizational activities) for those with little to no experience and collaborative (integration, higher formalization, parties work collectively through common strategies toward common goals) for those with more experience (Fig. 2). In addition to the understanding of the relational nature of the domains against degrees of collaborative experience, we were able to define the hierarchy against two of four dimensions on the Bailey and Koney [27] strategic alliance spectrum that serves as a means in which to understand the degree of informal/formal interactions between team members. Example scale results are included in the figure, with those scoring on the cooperative scale tending to display less experience and readiness for team engagement, while those scoring on the collaborative scale display more experience and readiness to collaborate. These are relative comparisons, however. In addition to this output score, one can also identify with the hierarchy of motivators for each scale, which differ in intensity in each scale. For example, building relationship motivations (dark blue) are a higher order motivator in collaborations than in cooperative engagements where they are more scattered across the hierarchy. For the participant who scores on the collaborative scale, these types of motivators proved to be more relevant to understanding readiness for someone engaging in teaming arrangements. The motivational “field” serves as a range of indicators that describes the motivations that are most specific to the participant taking the MATRICx.

Figure 2 explains two individual scenarios. Participant A scores highly on the cooperative scale, suggesting she or he has self-reported little to no previous collaboration experience, yet enough experience to include the motivators adjacent to the red line on the cooperative scale. In addition, by noting the specific items along the cooperative scale, one can see the motivators that are stronger for Participant A (namely the first 13 from the bottom up). In contrast, Participant F, who has more experience in collaboration and thus scores on the collaborative scale, though spanning less of the overall range of motivators, is more strongly motivated by the first six motivators on the scale along the green line (from the bottom up). It is critical to be continually aware that the cooperative scale is fixed and a prediction of those who self-reported as having less experience. The same needs to be stated for the collaborative scale. When we look closer at the actual motivators for each participant, we can see that Participant A spans almost all of the motivator domains suggesting that she/he have a variety of motivating factors related to a particular project or team at any given time. Participant F is more situated within a zone of higher order and more tightly focused motivations many of which are directed to motivations about advancing science. In addition, it should be noted that if one were to rate on the collaborative scale, the motivators fall into a more predictable domain pattern from advancing science, with knowledge transfer, and building relationships being higher on the scale. In comparison, Participants A and F are clearly separated by their self-reported historical experiences and the scale assists in identifying what motivators are more likely to need maximization and nurturing for team member satisfaction of needs.

## Results

An initial *paper and pencil* test of the original items that over time and testing were culled into the 49-item survey is now representative of the assessment. Through alterations resulting from pilot and cognitive testing, the finalized survey was digitized and developed into a live mobile application allowing participant, team, and composite results to be delivered to the user immediately upon completion of the survey. Those scoring on the cooperative scale tend to display less experience and readiness for scientific team engagement, while those scoring on the collaborative scale display more experience and readiness to collaborate scientifically.

**Fig. 3.**
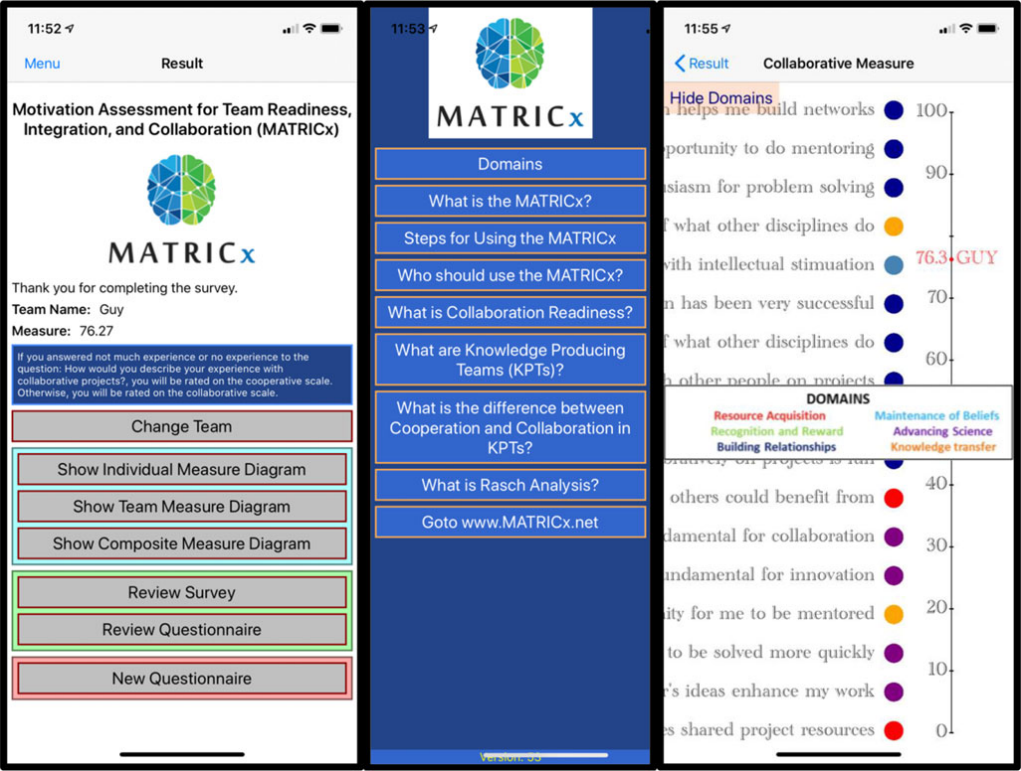
Visual panels of the MATRICx mobile application.

To manage the survey and output of the MATRICx, the team developed a mobile application available through Android and the Apple Store free to users interested in utilizing the survey in teaming exercises. Data collected is stored on REDCap and is de-identified for individuals and can be self-tagged for teams. Figure 3 displays some of the visual panels available to the user showing (from left to right) the participant’s landing page with controls and data archive, information about the components and construct of the MATRICx and the scientific support, and an example of one participant’s results who scored on the collaborative scale.

These results can be used to compare individuals’ readiness or to develop a profile for an entire team, which can be useful to team leaders and facilitators as they explore how to maximize motivations in a team. In addition, the application allows data to be compared to composite data of all MATRICx users. Outputs for the individual, team, and composite scoring are all accessible through the mobile application. To learn more about the application go to www.MATRICx.net.

## Discussion

Individuals join KPTs for various reasons, driven in part by intrapersonal motivation. The MATRICx is a vehicle for fostering individual reflection upon factors that motivate them to collaborate, which are often influenced by the amount and quality of their past experiences working with others.

For individual team members, the completion of the MATRICx assists to better identify their pre-conceived attitudes towards working as part of a team, focusing on motivations that can be exploited when exploring team roles and desired activities, that help team members assess their own readiness to collaborate, and note how it compares to other team members.

For KPT leaders, the MATRICx output provides an examination of the extent to which each team member is motivated towards cooperation versus collaboration within a science team, the common indicators influencing their motivations, a gauge by which to ensure that team member remain engaged, as well as the overall readiness of the team to work together. In addition, the leader can understand that the members are motivated by different reasons to collaborate.

For facilitators of teams and those charged with individual and team development, the MATRICx is valuable as a preparatory or ongoing tool to understand the motivations at play, assist leaders to strategize how to prepare and support teams based on their individual and team motivations, ensure ongoing engagement based on motivation and the satisfaction of needs, and create developmental activities that further explore and maximize motivations.

## Limitations

Utilization of the MATRICx requires self-reflection and reporting, as well as the voluntary participation and sharing of results by individual team members. This provides a limitation to the generalization of data in that individuals and teams are required to self-analyze the meaning of motivations as they pertain to specific contexts. This application of results to the team context is critical to benefiting from the assessment.

## Conclusions and Future Research

The MATRICx has potential practical value to KPTs who are attempting to understand team motivations so that these can be maximized to ensure greater individual and team performance of the team. We believe this will be especially useful in the creation and maintenance of scientific teams inclusive of multi-role and multi-discipline stakeholders. It can be used as a strategic engagement tool and has the potential for measuring longitudinal motivational changes within individuals and teams, especially amidst team membership changes, by providing a measure that is dedicated to understanding the motivations that impact the sharing of knowledge through collaborative research and scholarship. It is therefore useful in team building and assessing capacity for integration of knowledge, assessing team readiness and strategic development, onboarding new team members, and developing of activities and interventions designed to strengthen team bonds. Learning interventions can be designed to maximize motivating factors, as team leaders address individual and team needs. Identification of these needs can guide team leaders as they form and strive to sustain their teams. In addition, teaming and learning opportunities can be implemented to allow individual members to challenge and potentially change the attitudes that are influencing their motivation, ultimately enhancing their readiness to collaborate.

Early, it was noted that these initial indicators could be organized into a hierarchy, similar to Bailey and Koney’s Strategic Alliance Continuum model, which later informs our research and continual work on intervention strategies, and how this continuum might align with satisfaction of psychological needs as represented in Maslow’s hierarchy of need [26,27]. Presently this research is being conducted through a mixed methods study that triangulates MATRICx outputs against semi-structured interviews in teams of different focus: health policy, education, and biomedicine. The results of this research will provide a more robust interpretation of the domains and motivating factors based on team interactions and attitudes toward cooperation and collaboration. For use in other types of research, we recommend that the MATRICx be used in longitudinal studies with goals to map change in team structure and design based on changing motivations amongst existing team members and in teams with revolving team members. In addition, the results of the MATRICx can prove to be useful in identifying trends amongst those with lesser and higher experience in teams as a baseline for studying success of teams against readiness for team engagement. Lastly, the MATRICx can be a useful instrument in studies that strive to measure the delta between individual and team motivations and how these differences might contribute to bonding dynamics in teams like trust, psychological safety, and values associated with low to high formalization in teams as they relate to individuals in teams.

The MATRICx as an individual and team development mediator can be a highly useful tool for generating activities by group facilitators. While many facilitators working in science environments will possess the skills necessary translate the results of the MATRICx into their own developmental repertoire, the authors realize that this is not always going to be the case. Presently, the research team is working on a series of learning interventions to be used alongside the MATRICx tool that will coincide with each of the tool domains as they relate to the hierarchy of psychological need satisfaction [26]. This work is meant to pair knowledge about individual and team motivation with the psychological needs of team to maximize satisfaction in collaborative engagements. The learning interventions being created are directly targeted at building higher level satisfaction in team engagements. To learn more about the development of these learning interventions go to www.MATRICx.net.
